# Uveal effusion syndrome: a case report

**DOI:** 10.1186/s13256-024-04496-1

**Published:** 2024-03-21

**Authors:** Brittany Hodges, Felix Omoruyi, Karen Allison

**Affiliations:** University of Rochester School of Medicine and Dentistry, Rochester, New York USA

**Keywords:** Uveal effusion syndrome, Choroidal effusion, Non-rhegmatogenous retinal detachment, Sclera, Vortex vein, Angle closure glaucoma, Sclerectomy, Diamox, Alphagan, Dorzolamide, Pilocarpine, Lantoprost, Cosopt, Brimonidine, Prednisone

## Abstract

**Background:**

This case report is applicable to the field of ophthalmology because there is a paucity of medical literature related to the clinical presentation, diagnosis, and management of uveal effusion syndrome. This is an urgent concern because there are severe complications associated with this disease, including non-rhegmatogenous retinal detachment, angle closure glaucoma, and possible blindness. This report will fill clinical knowledge gaps using a patient example.

**Case presentation:**

A 68-year-old white male with multiple cardiovascular risk factors initially presented to the Eye Institute Urgent Care Clinic with new onset visual symptoms, including eye pain, eye lid swelling, redness, and tearing of his left eye. He had experienced a foreign body sensation in the left eye and bilateral floaters weeks prior to his presentation. The patient was examined, and vision was 20/30 in both eyes, and intraocular pressure was 46 in the right eye and 36 in the left eye. After initial assessment, including compression gonioscopy, intermittent angle closure glaucoma was suspected. He received oral diamox 500 mg, one drop of alphagan in both eyes, one drop of latanoprost in both eyes, one drop of dorzolamide in both eyes, and one drop of 2% pilocarpine in both eyes. There was only slight response in intraocular pressure. Owing to the bilateral angle closure, he underwent laser peripheral iridotomy to decrease intraocular pressure and open the angle that was found closed on gonioscopy. The patient was discharged on oral and topical glaucoma drops and scheduled for the glaucoma clinic. When he presented for follow-up in the glaucoma clinic, he was evaluated and noted to have bilateral narrow angles and intraocular pressure in the mid-twenties. A brightness scan (B-scan) was performed and was noted to have bilateral choroidal effusions, confirmed by Optos fundus photos. He was started on prednisone at 60 mg once per day (QD) with taper, continuation of oral and topical glaucoma medications, and a retina evaluation. Evaluation with a retina specialist showed resolving choroidal effusion in the left eye. He continued the prednisone taper as well as glaucoma drops as prescribed. Follow-up in the glaucoma clinic revealed a grade 3 open angle. He continued the prednisone taper, cosopt twice per day in both eyes, and discontinued brimonidine. The magnetic resonance imaging (MRI) that was performed showed results that were remarkable. No hemorrhage or mass was present. Follow-up with the retina specialist found that the choroidal effusions had resolved completely.

**Conclusion:**

This case report emphasizes the value in early detection, keen diagnostic evaluation, and cross-collaboration between multiple ophthalmology specialists to optimize healthcare outcomes for patients with uveal effusion syndrome.

## Background

Uveal effusion syndrome was first described in the medical literature by Schepens and Brockhurst in 1963. In 1982, Gass and Jallow hypothesized that the primary underlying cause of idiopathic uveal effusion syndrome is a congenital anomaly of the sclera, and in some cases, the vortex veins [1].

Idiopathic uveal effusion syndrome is a rare disorder that typically affects healthy middle-aged males with a relapsing–remitting clinical course. Presently, there are no known risk factors for the disease [7].

Uveal effusion syndrome can be classified into three subtypes:

- Type 1: nanophthalmic eye: small eyeball with an average axial length of 16 mm and high hypermetropic with an average of +16 diopters.
- Type 2: non-nanopthalmic eye with clinically abnormal sclera: normal eyeball size with an average axial length of 21 mm and small refractive error.
- Type 3: non-nanopthalmic eyes with clinically normal sclera [2, 5].

The pathogenesis of uveal effusion syndrome is thought to involve a primary choroid or scleral abnormality, which impedes transscleral intraocular fluid outflow. This leads to vortex vein compression and congestion of the choroidal veins resulting in intraocular fluid retention in the choroid. Serious complications involve non-rhegmatogenous retinal detachment.

## Case presentation

On 18 November 2022, a 68-year-old white male with multiple cardiovascular risk factors that included coronary artery disease, controlled hypertension, and hyperlipidemia presented to the Eye Institute’s Urgent Care Clinic with new onset visual distortions, pain, swelling, redness, and tearing in his left eye. For the week prior, the patient had experienced a foreign body sensation and a sharp pain that lasted about 5 seconds. For the previous 2 weeks, he had been experiencing new bilateral floaters. He endorsed headache that correlated with eye pain. He denied light flashes, scalp tenderness, fever, chills, pain or difficulty chewing food, or muscle weakness.

Initial eye exam revealed that vision was 20/50 in the right eye and 20/30 in the left eye extraocular movement (EOM) and were full, and pupils were normal with no afferent pupillary defect (APD). The intraocular pressure (IOP) was 46 in the right eye and 39 in the left eye with gonioscopy showing no angle structures in both eyes.

After initial assessment with tonometry, gonioscopy, slit lamp, and fundus exam, intermittent angle closure glaucoma was suspected in both eyes owing to elevated intraocular pressure, partially closed angles, and partial iris bombe configuration. The patient received oral diamox 500 mg, one drop of alphagan in both eyes (OU), one drop of dorzolamide OU, one drop of 2% pilocarpine OU, and one drop of latanoprost OU. There was only a minimal decrease in intraocular pressure. The patient underwent laser peripheral iridotomy where his intraocular pressure dropped to 26/27. He was then prescribed lantoprost once per day OU, cosopt twice per day (BID), brimonidine three times per day OU, and oral diamox of 500 mg BID, with a follow up in 1 week.

On 23 November 2022 the patient returned for a follow-up visit in the glaucoma clinic. Evaluation revealed that IOP was 20 in the right eye and 10 in the left eye and the slip lamp and fundus exam were unchanged. His angles were closed with a very shallow anterior chamber. Humphrey visual field testing showed arcuate scotoma in his left eye. A B-scan performed was notable for bilateral choroidal effusions. This was confirmed with Optos fundus photos. Medical management included initiating oral prednisone at 60 mg QD with taper (decreased by 10 mg every 5 days), latanoprost every night at bedtime, brimonidine three times a day, cosopt BID OU, and oral diamox at 250 mg BID with surgical drainage if no resolution. Magnetic resonance imaging (MRI) of the head and orbit was ordered with a plan to follow up with a retina specialist.

On 1 December 2022, the patient was evaluated by the retina specialist. He reported improvement of his visual acuity in his right eye; however, he endorsed persistent visual blurriness and floaters. His glasses were not helpful. He denied pain, flashes, burning, itching, or tearing. Evaluation showed resolving choroidal effusion in his left eye. Management involved continued prednisone taper (currently at 50 mg QD) and previous glaucoma drops as prescribed with a follow-up in the glaucoma clinic.

On 12 December 2022, the patient presented to the glaucoma clinic for follow-up with blurry vision in his right eye, intermittent sharp pain in both eyes and bloodshot eyes that started the week prior. He endorsed headache, increased pressure, and floaters. He denied flashes. He reported adherence to his ocular medications. Gonioscopy revealed that the angle increased to grade 3 in both eyes. He was continued with prednisone taper (currently at 30 mg QD), cosopt BID OU, and discontinuation of brimonidine. MRI results were remarkable. There was no evidence of hemorrhage, atrophy, mass, or fluid. He was followed-up with by the retina specialist on 13 December 2022; the choroidal effusions had resolved completely as well as the patient’s symptoms. He was scheduled to follow up in the glaucoma clinic for management of his secondary glaucoma.

## Discussion

This case presentation highlights the complexity in accurate diagnosis, efficient management and effective treatment for uveal effusion syndrome. Typical symptoms are non-specific and may include vision loss, blurred vision, or intraocular pressure that is either within normal limits or elevated. A clinical diagnosis may include angle closure glaucoma, creeping angle closure glaucoma, detachments of choroid and ciliary body, non-rhegmatogenous retinal detachment with shifting fluid, and minimal or absent evidence of uveal, retinal, or vitreous inflammation and localized areas of retinal pigmental epithelium hypertrophy and hyperplasia (“leopard spots”). Diagnostic procedures may entail a fluorescein angiography to rule out other causes of exudative retinal detachment with fluorescein dye or indocyanine green, B-scan ultrasonography, ultrasound bio microscopy to measure scleral thickness 2 mm and 3 mm posterior to the scleral spur, ultrasound biomicroscopy (UBM), or optical coherence tomography using Optos to evaluate the peripheral retina.

General treatment options for uveal effusion syndrome involve systemic steroids, surgical decompression of the vortex veins [4, 6], full thickness sclerectomies, and vitrectomy. The prognosis is generally favorable, with a large case series suggesting that sclerectomy produces an anatomic improvement in approximately 83% of treated eyes after a single procedure and 96% of treated eyes after one to two procedures. These studies show that final visual acuity improves by two or more lines in 56% of eyes, is stable in 35%, and worsens in 9% [3].

## Conclusion

This presented case of uveal effusion syndrome underscores the challenges in diagnosing, treating, and managing a rare ocular disorder. It also emphasizes the value in early detection, keen diagnostic evaluation, and cross-collaboration between multiple ophthalmology specialists to optimize patient care outcomes. Future discussions on the topic center around the utility of systemic and topical steroids for treatment. Some studies suggest that systemic steroids are not effective in the management of uveal effusion syndrome. Further discussion involves the role of surgical decompression for uveal effusion syndrome. Surgical decompression of the vortex veins has been described, though the most common treatment is full-thickness sclerectomies to provide choroidal fluid drainage. An exit of subchoroidal fluid can be performed by full-thickness sclerectomy or subscleral sclerectomy, with or without the application of mitomycin C [8, 9]. Continued research is needed to better assess and characterize optimal diagnosis and treatment strategies for patients with uveal effusion syndrome.

## Data Availability

Not applicable.

## Abbreviations

OU: Both eyes
OD: Right eye
OS: Left eye
